# Predictors of teenage pregnancy in Ethiopia: a multilevel analysis

**DOI:** 10.1186/s12889-019-6845-7

**Published:** 2019-05-17

**Authors:** Betelhem Eshetu Birhanu, Deresse Legesse Kebede, Alemayehu Bayray Kahsay, Abate Bekele Belachew

**Affiliations:** 1 Department of Public Health, College of Medicine and Health Sciences, Hawassa University, Hawassa, Ethiopia; 2 School of Public Health, College of Health Sciences, Mekelle University, Mekelle, Ethiopia

**Keywords:** Cluster, Enumeration area, Mixed-effect logistic regression model, Weighted

## Abstract

**Background:**

In Ethiopia, pregnancy, and childbearing begin at an early age. Teenage pregnancy has long-term implications for girls, their families, and communities. However, multilevel predictors of teenage pregnancy are not well studied yet. Several studies are focused only on the effects of individual-level characteristics but ignored the community level effect. This, in turn, could result in biased estimation of predictors of teenage pregnancy. Therefore, this study aimed to identify the individual and community level factors that determine teenage pregnancy in Ethiopia.

**Method:**

The data were extracted from the 2016 Ethiopian Demographic and Health Survey. The study included a sample from 645 clusters of 2679 (weighted) women aged 20–24 years. The data were collected using a two-stage cluster design that includes selection of enumeration areas as a first stage and selection of households as a second stage. A two-level mixed-effect logistic regression model was fitted to determine the individual and community level factors associated with teenage pregnancy.

**Result:**

The study revealed that 2134(79.6%) of women aged 20–24 years experienced pregnancy during their adolescent stage. Being sexually active before age 15[AOR = 7.9; 95%CI: 4.5, 13.8]; being married before age 15[AOR = 30; 9%CI: 16.7, 53.9] and being a rural dweller [AOR = 2.2; 95%CI: 1.4, 3.6] were positively associated with teenage pregnancy. A woman living in a community with a lower proportion of contraceptive users [AOR = 2.3; 95%CI: 1.5, 3.5]; had also a statistically significant association with teenage pregnancy.

**Conclusions and recommendation:**

Various factors at both the individual and community level determined teenage pregnancy. Therefore, the government should work on the prevention of early marriage, early sexual initiation and on improving the utilization of family planning in the community to protect them from pregnancy that occur at early age.

## Background

Teenage is a time of transition from childhood to adulthood. World Health Organization (WHO) defines the age group 10–19 years as adolescents stage [1]. This stage is a transitional period that requires special attention and sustained support. There are physical, emotional, mental and social changes that place their life at high risk [2]. Consequently, most adolescents exposed to unwanted pregnancy, casual sexual practices; rape; childbearing at an early age, high-risk abortion, HIV/AIDS and other sexually transmitted diseases [3, 4]. In addition, adolescents do not receive adequate information and services on reproductive health [4]. This situation makes the problems associated with teenage reproductive health serious and complex.In sub-Saharan African countries; the magnitude of teenage pregnancy accounted for 28%; which is higher compared with the world average of 6.5% [5, 6]. Half of all adolescent births occur in just seven countries, three of which are in Africa (Ethiopia, the Democratic Republic of Congo, and Nigeria) [5]. Childbearing begin early in Ethiopia, with having little knowledge and limited access to reproductive health services [7]. A descriptive study conducted using vital statistic report showed that; in Ethiopia 520,700 pregnancies occurred among adolescents with 15–19 years of age by 2008 [8].

Teenage pregnancy has a major impact with respect to its medical, social and economic implications [9]. It results in greater health risks for both the mother and the child [10]. In Ethiopia, according to the 2011 Ethiopian Demographic and Health Survey (EDHS) report; teenage female deaths that related to maternal causes holds 22% [10]. It is estimated that 9000 new fistula cases occur annually in Ethiopia and one in three occurred as they were under 20 [11, 12]. Unsafe abortion is also more prevalent among adolescents that result in higher deaths of women and about 2.5 million adolescents have unsafe abortions that result in 68,000 deaths each year [13, 14].

Babies of teenage mothers are more likely to be of low birth weight with the risk of associated long-term effects [13]. Mothers under the age of 18 experience a 60% greater chance that their child will die in the first year of life [15]. This report also showed that stillbirths and death in the first week of life are 50% higher among babies born to teenagers. According to the 2011 EDHS report; neonatal and infant mortality rates were higher among those born to adolescents compared with those born to adult mothers in Ethiopia [10]. Teenage pregnancy also limits and ends girls’ potential; they are taken out of school to be mothers, and they are more likely to be unemployed [3]. Teenage pregnancy could have also its own effect on population growth rates and the overall fertility level [10]. Therefore, knowing predictors of teenage pregnancy is important to prevent its medical, social and economic impact.Although several studies identified a range of factors that determine teenage pregnancy in Ethiopia and elsewhere [16–19], they are focused only on the effects of individual level characteristics. Therefore, they used analytical techniques that assume independence of individual observations. However, the individual observations have some degree of correlation within a cluster or community they belong because of common characteristics they share [20]. Consequently, ignoring this fact generally results in incorrect conclusions on the effects of associated factors on teenage pregnancy [21, 22]. Thus, this study was aimed to identify both the individual and community level factors affecting teenage pregnancy using multilevel modeling. Therefore, the importance of both communities’ and individuals’ effects on individual adolescents’ decision to become pregnant was considered hence the effects were estimated efficiently [21, 22].

Moreover, the sampling methodology of the survey (EDHS) covers the entire territory of the country and the data were well monitored throughout the survey time [10]. Therefore, the estimates obtained from the current study can accurately represent the actual situation in Ethiopia. The study finding helps policymakers and program planners to design and implement appropriate programs and strategies at both individual and community levels to decrease the prevalence of teenage pregnancy. It can give direction about the current policies and programs being implemented on adolescent reproductive health in Ethiopia.

## Methods

### Study area

The study was conducted in Ethiopia which is a developing country, whose economy is entirely dependent on agriculture. Total population of the country was projected to be 102.3 million in 2016, with 83% living in rural areas [19]. Contraception use is the major preventive method for teenage pregnancy, but only 5.2% of married and sexually active teenagers use modern method of contraception in Ethiopia [10].

### Data source and study population

We have used birth data set of EDHS 2016 for this study. The data set was accessed from the Measure DHS website (http://www.measuredhs.com). To conduct the 2016 EDHS, a two-stage stratified cluster sampling technique has been employed. In the first stage, enumeration areas were selected. Enumeration area is a geographic area consisting of a convenient number of dwelling units which served as a counting unit for the census. In the second stage, 28 households per enumeration area were selected with an equal probability systematic selection per enumeration area [23].

To reflect the community level effect; 645 clusters or enumeration areas were included in this study. A total of 2679 younger women aged 20 to 24 years who were interviewed for age at first birth at the time of the survey were included in this study. These age groups were selected due to full exposure to the risk of pregnancy before age 20, and those beyond 24 years of age were not included because they are more likely to suffer from memory lapse and event omission compared with younger females.

Important variables were selected by referencing the DHS recode-6 manual and questionnaire at the end of EDHS 2016 report. Furthermore, selections of households, validation procedure anddata quality assurance are available in detail elsewhere [23].

### Study variables

#### Outcome variable

##### Teenage pregnancy

Teenage pregnancy: It is a composite binary outcome variable that refers to pregnancy experience of a woman before age 20 years. It was derived by concatenating age at first birth with age of the women at the time of the survey. Then it was categorized in such a way that 0 = no pregnancy before age 20 (that includes those who had their first pregnancy at age 20 or later, or never had pregnancy) and 1 = pregnancy experienced before the 20th birthday.

#### Independent variables

In this study, a two level variables (individual and community level) were considered as independent variables.

### Individual level variables

#### Age at first marriage

Age at first marriage is defined as the age at which the respondent began living with her first spouse/partner. It was encoded into three categories. “Married before age 15”, “married at age 15-17” and “not married before 18”. Not married before 18 years of age includes those who had been married at age 18 and after; or were not married in their age of life.

#### Sexual experience

Sexual experience was encoded into three categories: “active before age 15”, “active at age 15-17” and “active at the age of 18 and above”.

#### Educational status of women

This variable has categories of “no education”, “primary”, “secondary” and “higher” in the 2016 EDHS. In the current study, provided a small number of cases in the higher category, it was merged to secondary level hence recoded to no education, primary and secondary or above categories.

#### Employment status of women

In the EDHS; employment status related data was collected as “no job” or as a list of different jobs. For this study, those lists of jobs were merged together and a dichotomized variable was generated as“ employed” and “not employed” regardless of the type of employment.

#### Media exposure

Watching television (TV), listening to radio and reading newspaper at least once a week were considered to measure exposure to media for both women and their partners’ in the 2016 EDHS. But the reading newspaper was not included in the current study because according to the 2016 EDHS most women (95%) in Ethiopia have no exposure to print media [10]. Therefore, for this study reading newspaper was not included. Consequently, a new variable was generated by concatenating the other two media sources (TV and Radio). The categories include: “Both at least once a week”, “Either at least once a week” and “No accesses at least once a week”.

#### Wealth index

The 2016 EDHS categorized wealth index with the national-level wealth quintiles (from lowest to highest) [10]. This variable was derived from the different assets of the households to assess the household cumulative wealth status. In the dataset, the categories for wealth index were presented as Poorest, Poorer, Middle, Richer, and Richest. In this study, by merging poorest with poorer and richest with richer a new variable was generated with “Poor”, “Middle” and “Rich” categories.

#### Religion

In the 2016 EDHS, religion has subcategories of Orthodox, Muslim, Protestant, Catholic, traditional followers and others. Since the former three were dominant of other religions with their frequency, they were encoded independently. Given that few number of Catholic and traditional religion followers, they merged to others.

### Community level variables

Community-level variables were generated by aggregating the individual level data into cluster except for place of residence and geographical region that were taken as it is. In the 2016 EDHS, place of residence was one of the characteristics that helped in designing the sample to give population and health indicators at the national level. Region was the geographical location where population lives in that directly explain the community characteristics. It indicates the 11 administrative regional states of Ethiopia namely Tigray, Afar, Amhara, Oromiya, Somali, Benishangul-Gumuz, Southern Nations, Nationalities and Peoples (SNNP), Gambela, Harari, Addis Ababa and Dire Dawa. All of them were encoded independently.

Other community-level variables were obtained by aggregating the individual women characteristics into clusters. They were computed using the proportion of a given variables’ sub category we were concerned upon per cluster. Since the aggregate values for all generated variables have no meaning at the individual level, they were categorized into groups based on the national median values. Median values were used to categorize as high and low because all the aggregated variables were not normally distributed. Similar procedures were applied to all aggregate variables. Community contraception use was generated based on the proportion of women who ever use family planning in the clusters. It shows the overall family planning utilization in the community. Community unmet need for family planning (supply) was also created based on the proportion of women with unmet need for family planning in each cluster. Community educational status was also generated based upon the proportion of educated community in each cluster. It shows the overall female educational attainment in the community. Community poverty status was created based on the proportion of poor women within their cluster. Community media exposure was produced based on the individual response for exposure to radio and TV.

### Statistical analysis

#### Multilevel regression analysis

In data with a nested structure like that of EDHS, the individual observations have some degree of correlation within a cluster because of common characteristics they share. As a result, when the correlation with the upper level is ignored and only the individual level characteristics are considered, it might lead to a violation of the assumption of independence between observations. This result in biased parameter estimates and will generally lead to underestimation of the standard errors and, produce spurious significant results and accordingly to incorrect conclusions on effect sizes [21]. In contrast, modeling group-to-group variation simultaneously with individual-to-individual variation in analysis has several advantages. It allows us to focus on the importance of both communities’ and individuals’ effects on individuals’ health outcome. By using the clustering information, it enables us to obtain statistically efficient estimates of regression coefficients [21, 22]. Therefore, to get the mixed effect (fixed effect for both the individual and community level factors and a random effect for the between cluster-variation), a two-level mixed-effect logistic regression analysis was used in this study. Thus, the log of the probability of being pregnant to a teenage was modeled in the following form;

$$ \log \left[\frac{\uppi_{\mathrm{ij}}}{1-{\uppi}_{\mathrm{ij}}}\right]={\upbeta}_0+{\upbeta}_1{\mathrm{X}}_{\mathrm{ij}}+{\upbeta}_2{\mathrm{Z}}_{\mathrm{ij}}+{\mathrm{u}}_{\mathrm{j}} $$

Where, i and j are the level 1 (individual) and level 2 (community or clusters) units, respectively; X and Z refer to individual and community-level variables, respectively; π_ij_ is the probability of being pregnant for the i^th^ teenager in the j^th^ community; the β’s are the fixed coefficients-therefore, for every one unit increase in X/Z (a set of explanatory variables) there is a corresponding effect on the probability of being pregnant to teenager. Whereas,β_0_ is the intercept –the effect on the probability of being pregnant to a teenager in the absence of influence of predictors; and u_j_ shows the random effect (effect of the community on a teenager decision to become pregnant) for the j^th^ community.

The clustered nature of the data, and the within and between community variations were taken in to account by assuming each community has different intercept (β_0_) and fixed coefficient (β).The amount of community variation was expressed as Intra-class Correlation Coefficient(ICC) computed as; $\mathrm{ICC}=\frac{\updelta^2{\mathrm{u}}_0}{\updelta^2{\mathrm{u}}_0+\frac{\uppi^2}{3}}$ where, $\frac{\pi^2}{3}$ denotes the variation within a cluster (individual level) and *δ*^2^u_0_ is the variation between clusters (communities).

We analyzed the data using STATA 12 (Stata Corp. Inc. Texas, USA). Since the sampling procedure for EDHS 2016 was complex; the selection procedure deviates from the assumption of randomness (simple random selection). To account this problem, the data were declared for its complexity and the variables were designated with information about the survey design before analysis. Moreover, sampling weights were done to proceed with the descriptive statistics such as frequency and proportions to adjust for non-proportional allocation of the sample to strata (urban and rural dwellings) and regions during the survey process.

Various model diagnostics were done for the final model. The study considered several factors which might modify the effect of each other on teenage pregnancy, the interaction effect was checked and there was no interaction effect between the covariates. Moreover, the multicollinearity (correlation of predictors with each other) was checked by using variance inflation factors (VIF) and no variable had VIF greater than 5, indicated the absence of significant collinearity among explanatory variables. Akaike’s Information Criterion (AIC) was used to choose a model that best explains the data and the model with low AIC value was taken. A test of how well the model explains the data (goodness of fit test) was checked by using Hosmer-Lemshow statistics and it was non-significant (prob> chi^2^ = 0.1270), indicating the model fits the data reasonably well. The predicting ability of the model (model accuracy) was evaluated using the Receiver Operating Characteristic (ROC) and it was 85.03%, indicating the model was good enough in differentiating pregnancy experienced from non-pregnancy experienced subjects correctly [24].

Statistically significance association was tested using Wald statistics, with results *p*-values less than 0.05 were considered statistically significant. The results of fixed effects were presented as adjusted odds ratio (AOR) at their 95% confidence interval (95% CI).

## Results

### Background individual and community level characteristics of study participants

Data from a weighted sample of 2679 women aged 20–24 years were included in this analysis, of whom 2134 (79.6) experienced pregnancy before age 20 years. Eight hundred eighty-three (33%) women had their first marriage and 800 (30%) had their first sex before age 15 years. Women who had no formal education were 1257(47%). According to the EDHS wealth index estimate, 1370 (51%) of the women were poor (Table 1).

**Table 1:** Teenage pregnancy by the Individual and Community level background characteristics among the study population (*n* = 2679), Ethiopia 2016

Background characteristics	Teenage pregnancy, %	Total; n (%)
Individual women characteristics		
Contraception use		
Yes	74.74	883(33)
No	83.29	1796(67)
Age at first marriage		
Married before 15	96.82	883 (32.95)
Married at 15–17	92.12	1161(43.35)
Not married before age 18	38.2	635(23.70)
Sexual experience		
Active before 15	96.29	800(29.87)
Active at 15–17	90.64	1277(47.68)
Active at age 18 and above	42.71	602(22.45)
Educational status of women		
No education	87.57	1257(46.94)
Primary	79.45	1164(43.46)
secondary or above	61.68	257(9.60)
Employment status of women		
Employed	78.26	933(34.84)
Not employed	82.26	1746(65.16)
Wealth index		
Poor	85.44	1370(51.13)
Middle	81.89	535(19.97)
Rich	72.35	774(28.90)
Media exposure		
Both at least once a week	68.97	101(3.78)
Either at least once a week	71.94	399(14.90)
No accesses at least once a week	83.45	2179(81.32)
Community level characteristics		
Place of residence		
vUrban	67.3	225(8.57)
Rural	84.01	2403 (91.43)
Community wealth status		
Lower proportion of poor	75.26	1491(55.66)
Higher proportion of poor	85.44	1188(44.34)
Community media exposure		
Higher proportion have exposure	77.45	1281(47.81)
Lower proportion have exposure	84.33	1398(52.19)
Community contraception use		
Higher proportion of users	73.4	1540(57.50)
Lower proportion of users	86.55	1139(42.50)
Community unmet need for family planning		
Higher proportion with unmet need	83.35	1749(65.28)
Lower proportion with unmet need	78.52	930(34.72)
Community educational status		
Higher proportion of educated	76.35	1314(49.05)
Lower proportion of educated	85.36	1365 (50.95)

In this study, 645 clusters were included. While measuring the characteristics of the community in those clusters, 2403 (91.4%) of the women were from rural clusters. From the total study subjects 1398(52.2%) were from lower community media exposure and 1139 (42.5%) were from a lower proportion of contraception user community. Around two-thirds of the women 1749(65.3%) were living in communities with a higher proportion of unmet need for family planning. About half (51%) of them were living in clusters with lower educated community (Table 1).

### Individual and community level predictors of teenage pregnancy

In the crude multilevel modeling, contraception use, age at first marriage, sexual experience, media exposure, place of residence, community wealth status, community media exposure, community contraception use status, community unmet need of contraception and community educational status were tested for an association with teenage pregnancy. All of those variables had shown statistically significant association (Table 2). Educational status, occupational status and wealth status of women were not tested because while measuring these variables, characteristics at the time of the survey might not be exactly the same with characteristics when young girls encountered pregnancy. On the other hand, these variables can be potential consequences instead of being factors to determine teenage pregnancy.

**Table 2:** The individual and community level factors associated with Teenage Pregnancy in Ethiopia, 2016 (n = 2679)

Background characteristics	COR[95%CI]	AOR[95%CI]
Contraception use		
Yes	1.00	1.00
No	1.4^*^[1.1, 1.9]	0.8[0.5, 1.2]
Sexual experience		
Active before 15	73.1^***^[46.01, 15.9]	7.92^***^[4.6, 13.8]
Active at 15–17	18.9^***^[12.9, 27.9]	3.5^***^[2.3, 5.5]
Active at age 18 and above	1.00	1.00
Age at first marriage		
Married before 15	137.7^***^[82.7, 229.4]	30.1^***^[16.8, 53.9]
Married at 15–17	33.3^***^[21.4, 51.8]	15.1^***^[9.2, 24.6]
Not married before age 18	1.00	1.00
Place of residence		
Urban	1.00	1.00
Rural	3.5^***^[2.4, 5.3]	2.2^**^[1.4, 3.6]
Community wealth status		
Lower proportion of poor		
Higher proportion of poor	2.5^***^[1.7, 3.6]	1.0[0.6, 1.8]
Community media exposure		
Higher proportion have exposure	0.5^***^[0.3, 0.7]	0.9[0.6, 1.5]
Lower proportion have exposure	1.00	1.00
Community contraception use		
Higher proportion of users	1.00	1.00
Lower proportion of users	3.1^***^[2.2, 4.4]	2.3^***^[1.5, 3.5]
Community unmet need for family planning		
Higher proportion with unmet need	1.7^**^[1.2, 2.4]	0.9[0.6, 1.4]
Lower proportion with unmet need	1.00	1.00
Community educational status		
Higher proportion of educated	1.00	1.00
Lower proportion of educated	2.4^***^[1.7, 3.4]	0.78[0.5, 1.2]

*AOR* Adjusted Odds Ratio,*CI* Confidence Interval;Significant at:^***^P < 0.001^,**^P < 0.01,^*^P < 0.05.

In the two-level mixed effect multivariable logistic regression model where both the individual and community level factors were fitted simultaneously; the odds of experiencing pregnancy at teenage was 7.9 times [AOR = 7.9; 95%CI: 4.6, 13.8] higher among women who were sexually active before age 15 compared with women who were sexually active at the age of 18 and above after holding all other predictors constant. A woman who was married at an early age (before 15 years) had 30.1 times [AOR = 30.1; 95%CI: 16.8, 53.9] higher odds of experiencing pregnancy at an early age compared with a woman who was not married before the legal age of 18 years.

A woman who was living in the rural area had 2.2 times higher odds of experiencing teenage pregnancy compared with a woman who was living in urban area [AOR = 2.2;95%CI:1.4. 3.6].

Community contraception use was another predictor of teenage pregnancy. A woman living in community with lower proportion of contraception use had 2.3 times [AOR = 2.3; 95%CI: 1.5, 3.5] higher odds of experiencing pregnancy at an early age compared with a woman living in clusters with a higher proportion of contraception use (Table 2).

### Random effect results

In the null model, the ICC value was $\left(\mathrm{ICC}=\frac{\updelta^2{\mathrm{u}}_0}{\ {\updelta}^2{\mathrm{u}}_0+\frac{\ {\uppi}^2}{3}}=\frac{2.34}{2.34+3.29}=0.416,\mathrm{P}<0.001\right)$. This.

indicates that about 42% of the total variation on teenage pregnancy occurred at the community level and is attributable to the community level factors. The existence of greater than zero ICC in the null model indicates that we did better in using multilevel modeling than the standard single-level regression model. However, after taking both the individual and community level predictors into account (i.e. in the combined model), the community level variability has been reduced to 38%. The model also showed the highest Proportional Change in Variance (PCV); that is 13%, indicating 13% of the community level variation on teenage pregnancy was explained by the combined factors at both the individual and community levels (Table 3).

**Table 3:** Community level variance of two-level mixed effect logit models predicting Teenage pregnancy, Ethiopia 2016

Random effect	Null model	Final model
Community level variance	2.34	2.03
ICC (%)	42	38
PCV (%)	Reference	13
Model fitness statistics(AIC)	2573.10	1581.77

***ICC*** Intra-community Correlation Coefficient, ***PCV*** Proportional Change in Variance, ***AIC***-Akakian Information Criteria, ***Null model*** model without any predictor, ***Final model***- A model where both individual and community level variables were fitted simultaneously

## Discussion

According to this study, more than three-fourths (79.6%) of the study population had experienced pregnancy before age 20 years. Several factors at both the individual and community levels were identified with a significant effect on teenage pregnancy. At the individual level (for instance, early marriage and early sexual experience); and at the community level; a lower proportion of community contraception users had higher teenage pregnancy experience.

The proportion of teenage pregnancy was comparatively higher than findings reported from Western Nigeria (22.9%) [25], South Africa (19.2%) [26], Uganda (6.5%) [27], Zambia (11.67%) [28], Portugal (51.5%) [29] and Bangladesh (72.5%) [18]. This might happen due to the difference in the demographic and socio-cultural characteristics of the countries such as marital age, age at sexual initiation and family planning utilization.

Early sexual initiation amplified early pregnancy and this is consistent with other studies conducted in Cameroon, South Africa and Nicaragua [17, 26, 30]. This might happened due to limited utilization of contraception and having limited knowledge on how to prevent pregnancy that happens before maturity. Most prevention efforts do not reach these teens; leaving them without the necessary skills and information to become a responsible decision maker about their sexual behavior [4]. Various studies showed that in most communities of Ethiopia, cultural beliefs within the community prevent teenagers from gaining more information about sexuality [31, 32]. Culturally, parents could not discuss sexual matters with their children. Usually, concrete sexual education and related information are not available for adolescents until they are faced with the trauma of unwanted pregnancy and other related complications.Early married women had a higher likelihood of experiencing teenage pregnancy when compared with women who were not married before the legal age of 18. Adolescents who were married at an early age might not have enough information on the risk of premature pregnancy and may not want to prevent untimely pregnancy. Married teenagers tend to miss better access to reproductive health services [33] and have limited knowledge of the use of contraception [34]. Cultural barriers might also influence their decision on contraception use. Their thought regarding need is mostly determined by the adults in their marital families who often encourage early fertility [33]. Fear of side effect on contraception use and having limited education might also influence them on postponing their pregnancy time.

Even though there is a law that prohibits marriage before age of 18 in Ethiopia; most of the time it is not enforced and practically not applicable in most communities especially in rural areas [35, 36]. Early marriage is preferred by families for different reasons typically in rural areas. Families prefer to get their daughters married while they are alive or before they get old. They also need to fulfill their interest in accomplishing marriage with a wealthy family in order to improve their living conditions. Besides, families want to prove a bride from a decent family and ensure that the bride is married at the right and socially accepted age limit [2, 37].

In addition to the individual, characteristics of the community where the individuals are nested in also were explored in this study. Lower contraception use rate within the community significantly increases the probability of experiencing teenage pregnancy. The trustworthiness of this finding is assured by its consistency with an ecological study conducted in Portugal [38] and another study carried out in Cameron [17]. The association can be accepted because if the community has a lower proportion of contraceptive users, adolescents within that community might not get enough information from their neighbors on how and when to use family planning. In addition, they are more deficient in information on how to gain a family planning service from health facilities.

The main methodological strength of the study is the use of multilevel modeling technique. This helps to hold the fixed effects of both the individual and community level factors and the random intercept to explain the between-cluster differences concurrently. Due to the presence of limited variables that describes community characteristics directly in the EDHS; other derived variables were generated by aggregating the individual women characteristics. This intern helped us to assess the association of those community characteristics with teenage pregnancy in addition to the individual characteristics.

The 2016 EDHS is a nationally representative survey. To strengthening its representativeness; modifications have been done on the data like weighting in descriptive statistics. Therefore, the findings obtained from the current study can be generalized to the entire country owing to the use of representative data.

However, the study has some limitations. As any cross-sectional study, causality was not possible to establish for the factors dealt in the study because it is difficult to know which comes first; the exposure or the outcome variable. In addition to that, the study heavily depends on information pertaining to the timing of events like age at first marriage and age at sexual initiation. Therefore, recall bias might occur due to memory lapse. On the other hand, while measuring data on some variables like educational status and wealth index; characteristics at the time of the survey might not be exactly the same with characteristics when younger women encountered teenage pregnancy. Of course, to minimize this risk, only younger women were chosen as study participants instead of all women with the childbearing age group. Furthermore, EDHS data does not include information on family characteristics, parental rearing style, and cultural practices. Data of this nature would give a deeper understanding of why some adolescents engage in early sexual activity and how it is linked to teenage pregnancy.

## Conclusions and recommendation

When we conclude our finding, teenage pregnancy is higher in Ethiopia compared with sub-Saharan African countries as a whole and it’s world average size. The multilevel modeling approach used in the study allowed us to identify several factors at both the individual and community level that are associated with it. At the individual level, early sexual experience and early marriage have shown a significant positive association with teenage pregnancy. At the community level, being rural dweller and a lower proportion of contraception users in the community significantly increased the likelihood of experiencing teenage pregnancy. Hence, factors driving teenage pregnancy are various. Therefore, it requires multifaceted intervention strategies. The government should, therefore, focus on educating the community on the prohibition of early marriage and early sexual initiation. In addition to that, the government should work on improving the utilization of family planning in the community to protect them from early pregnancy.

### Ethics approval and consent to participate

The data were accessed from the Demographic and Health Survey (DHS) website (http://www.measuredhs.com) after getting registered and permission was obtained. The accessed data were used for the purpose of the registered research only. The data were treated as confidential and no effort was made to identify any household or individual respondent. Moreover, the EDHS approved by the Ethiopian Health Nutrition and Research Institute (EHNRI) Review Board and the National Research Ethics Review Committee(NRERC) at the Ministry of Science and Technology, Ethiopia. Further, as published in the survey report of 2016, verbal informed consent was obtained from all participants after the purpose of the research was explained [25]. Participation in the survey was voluntary as participants could decline to participate even after giving consent.

## Abbreviations

AIC: Akaike’s Information Criterion
DHS: Demographic and Health Survey
EDHS: Ethiopia Demographic and Health Survey
ICC: Intra-class Correlation Coefficient
PCV: Proportional Change in Variance
VIF: Variance inflation factor
